# The Rheumatic Diseases

**Published:** 1905-06

**Authors:** 


**The Rheumatic Diseases.** By J. ODERY SYMES, M.D. Pp. viii., 233. London: John Lane. 1905.

This newest work on rheumatic affections is founded entirely on the belief that rheumatic conditions are incited by a specific micro-organism, the diplococcus rheumaticus. It is further maintained that in the chronic forms of rheumatism, and in acute and chronic rheumatoid arthritis, the exciting cause is a modified or a virulent form of the coccus of acute rheumatism. The arthritis associated with many acute infective processes is also believed to be due to the action of a specific organism or its toxin upon the joint tissues: accordingly, gonorrhœal and scarlatinal arthritis are still included under the heading of rheumatic diseases, although the organism may not be that of acute rheumatism. No new classification is suggested. Six chapters make up the volume: acute rheumatism, the rheumatism of childhood, muscular and chronic articular rheumatism, acute and chronic rheumatoid arthritis, gonorrhœal rheumatism, and scarlatinal rheumatism.

The principal facts in the epidemiological and the clinical history of rheumatic fever point to the infective nature of the disease, and cases of direct infection have been recorded. The recent researches of many observers agree that the diplococcus of Wassermann may be isolated from the urine and sometimes from the blood of rheumatic cases, and may by inoculation into rabbits and monkeys produce various lesions identical with those found in rheumatic fever. The clinical picture of the disease is terse but comprehensive, and concludes with the remark that "a routine examination of the heart should be made by the medical attendant every six months, whether there have been previous endocarditis or not."

The chapter on the rheumatism of childhood includes a description of the frequent manifestations of the rheumatic state in childhood, the subcutaneous nodules, and chorea. The author would impress upon parents the necessity for careful watching of such children, and thinks they should be examined every three or four months periodically up to the age of puberty.

It is difficult to understand that muscular and chronic articular rheumatism should be due to the action of the same diplococcus which causes the acute conditions, nevertheless the author states that "by chronic rheumatism we understand an inflammation and hyperplasia of fibrous tissue, especially the fibrous tissue of the muscles, fasciæ, tendons, ligaments, and nerve sheaths, giving rise to pain, and which is caused directly or indirectly by the action of a specific organism, the rheumatic diplococcus." He thinks that although the condition may be the result of the direct action of the rheumatic diplococcus in the tissues, yet it is more probable that the changes are brought about by the rheumatic toxin. "Bacteriological researches have shown that the diplococcus may assume a quiescent form, and such a form lying latent in the bone-marrow, tonsils, fauces, endocardial vegetations, or joints may form a toxin which, under favourable circumstances, is capable of exciting a fibrositis."

The chapter on acute and chronic rheumatoid arthritis is one of the best. The synonyms fibro-arthritis for the former and osteo-arthritis for the latter are used, indicating that the two conditions have little in common. We think, however, that there is little connection between the monarticular arthritis of old people and the subacute or chronic polyarticular nodose arthritis of the middle-aged. This latter condition seems to be an intermediate link, and well deserves the separate designation, "chronic rheumatoid arthritis," as distinguished from osteo-arthritis proper. A point which the author insists on as of great importance is the "seeking out of a primary focus of infection, and the correction of the fault when found." This is the first and most important step in the treatment of rheumatoid arthritis.

The chapter on gonorrhœal rheumatism again emphasises the infective nature of the disease, inasmuch as "there is now a sufficient mass of evidence to enable us to state that this form of arthritis is caused by a general systemic invasion by the gonococcus, and that it is not to be regarded merely as an attack of rheumatism complicating gonorrhœa, or as a pyæmia due to one or more of the many other pyogenic organisms present in the urethral discharge."

As regards the last chapter, we learn that "all observers are agreed, however, that scarlet fever is an acute infective process, and we must regard scarlatinal arthritis as one of the direct consequences of the scarlatinal toxin."

The book has twenty-three excellent illustrations, mostly photographic plates; it is printed in excellent type, is therefore easy to read; is not encumbered with too great detail, and is singularly free from printers' errors. It gives the views of a thoughtful observer in an interesting although somewhat chaotic field, in which there is still scope for further investigation, and we trust that the author will be encouraged by this effort to continue his researches.

